# Dietary self-care and associated factors among diabetic patients in Jimma University Medical Centre, South West Ethiopia; A path analysis

**DOI:** 10.1371/journal.pone.0273074

**Published:** 2022-08-24

**Authors:** Musa Jemal, Alemayehu Argaw, Abonesh Taye, Tsion Sintayehu, Shemsu Kedir

**Affiliations:** 1 Departement of Public Health, College of Medicine and Health Science, Werabe University, Werabe, Ethiopia; 2 Department of Nutrition and Dietetics, College of Health Science, Jimma University, Jimma, Ethiopia; 3 Departement of Public Health, College of Medicine and Health Science, Wachemo University, Hosanna, Ethiopia; Polytechnic Institute of Coimbra: Instituto Politecnico de Coimbra, PORTUGAL

## Abstract

**Background:**

Diabetes Mellitus (DM), a chronic metabolic disorder that caused about 4.2 million deaths and at least 760 billion dollars’ expenditure in 2019, has been targeted for action by leaders of WHO member countries. In Ethiopia deaths, due to DM reached 34,262 in 2013. Studies show effective lifestyle interventions; particularly medical nutrition therapy reduces HbA1c by 0.5 to 2%. However, practicing recommended diet is reported to be difficult. Not only Knowledge and practice but also perception studies are therefore necessary to design future health programs.

**Objective:**

To assess diabetic self-care, dietary practice and associated factors among diabetes patients.

**Method:**

Institution-basedbased cross-sectional study design was employed from february15-May15, 2020 in Jimma university medical Centre (JUMC). Systematic sampling of every other patient (K = 2.7) was employed to interview 371 participants. A previously validated tool was used to collect data through a face-to-face interview. A path analysis was used to fit the structural model and tests the hypothesized Health Belief Model (HBM) relationships.

**Result:**

Response rate was 95.4% (354). Around 52% of the participants were male and 76.8% follow diabetic education at least some times. 42.4% and 48% of respondents have good dietary and general self-care practices respectively. With unstandardized coefficient (standard error) self-efficacy0.10 (0.01) being the strongest cues to action0.10 (0.02), perceived threat0.02 (0.01), and perceived barrier-0.08(0.01) constructs of HBM have a significant effect on dietary practice. Knowledge, social support and diabetes distress exert a significant indirect effect on dietary practice through health belief constructs with unstandardized path coefficient (standard error) of 0.22(0.03), 0.02(0.01), and -0.03(0.004) respectively.

**Conclusion:**

In this study, the proportion of good practice is found to be lower for both dietary as well as general self-care. HBM can best fit to explain variability in dietary self-care practice; therefore, future interventions should be designed to address the vast perception and psychosocial factors influencing dietary self-care practices.

## Introduction

American Diabetes Association(ADA) defines diabetes mellitus as“a general term for a group of metabolic disorders with disrupted carbohydrate, fat, and protein metabolism that results from defects in insulin secretion, insulin action, or both commonly characterized by elevated blood glucose” [1]. Currently ADA recommends a diagnostic criteria for diabetes that is Hgb–A1C > = 6.5%, or fasting blood sugar ≥ 7.0mmol/l (126mg/dl) or 2–hours plasma glucose tolerance test ≥ 11.1mmol/l (200mg/dl) or a random blood sugar ≥ 200 mg/dl (11.1 mmol/l) in patients with classical symptoms [2].

Though diabetes and other NCDs were considered a problem in developed countries, however, the increment is faster in low and middle-income countries than in high-income countries [3]. In the IDF Africa region,19million adults were living with diabetes in 2019, which is estimated to increase to 47 million by 2045. Preceded by South Africa, Nigeria, and DRC in decreasing order Ethiopia is among the top five African countries having 1.7 million patients living with diabetes in 20–79 year old adults by the year 2019 [4].

A constantly rising treatment cost for diabetes obliges developing countries, where the resources are limited, to practice diabetic self-care components, in which patients or their families usually carry out about 95% of the disease management, to have better economic and therapeutic outcomes [5]. Self-care practice includes performing well-recognized and specific self-care component activities in multiple domains to prevent and/or delay complications and the possibility of early death associated with diabetes. The components are self-monitoring of blood glucose level, diet control, optimum physical exercise, adherence to medication/s, and proper foot care [6, 7].

Effective lifestyle interventions that include a healthy diet and exercise can reduce diabetes incidence by up to 55% and have shown to be more efficient than antidiabetic medicines [8]. Of the essential lifestyle modifications for diabetes management, dietary modification is considered d to be of the cornerstone [9]. Different studies showed that Medical Nutrition Therapy(MNT) reduces the HbA1c by 0.5 to 2% [10] However, practicing and adhering to the recommended diet is reported by both health professionals and patients to be the most difficult among diabetes self-care areas [11].

Despite strategies that Ethiopia developed and endorsed such as the National Strategic Action Plan (NSAP) in 2016 [12] diabetes prevalence as well as morbidity and mortality associated with diabetes and its complication is increasing.

It has been suggested that psychological interventions may better improve glycemic control if they address an individual’s understanding, perception of control, and perception of the chronic nature of the disease [13, 14]. Not only Knowledge and practice studies but also perception studies are therefore necessary to design future programs and techniques for effective health education and promotion programs [15].

Although such studies are important in resource-limited areas; available researches either lack psychosocial dimensions that affect self-care activities greatly or an appropriate statistical analysis method as hypothesized by the model. Therefore, this study aims to show the level of dietary practice along with factors associated with it including patients’ perception of diabetes and dietary self-care.

## Methods and materials

### Study design and setting

Institution based cross-sectional study was carried out from February 15 to May 15, 2020 G.C in Jimma University Medical Centre(JUMC). JUMC is located in Jimma City, 357 km Southwest of Addis Ababa. It serves as a referral center for the Southwestern part of Ethiopia. DM follow up service is delivered on two days per week on Monday and Tuesday. There are 3038 patients living with diabetes registered in this clinic.

### Population of the study

The source populations were all patients living with DM who attend follow up service at JUMC chronic care clinic and the study populations were DM patients who visited JUMC diabetes outpatient department clinic during data collection period and fulfilled the study inclusion criteria.

### Eligibility criteria

A patient was included in the study if he/she was ≥18 years of age and had been receiving follow up services for at least 6 months at the time of data collection and consented to participate in the study. Patients with mental disorder, who were unable to communicate with the interviewer, patients with hearing impairments or any other serious health problems (acute diabetes complication) during data collection period were excluded from the study.

### Sample size and sampling technique

#### Sample size determination

Sample size was determined using single population proportion formula taking proportion of good dietary practice taken to be 44.3% from previous study [16], and margin of error which is 5%, with 95% confidence level. The final sample size became **371,** after adding 10% non-respondent rate.

#### Sampling procedure

Systematic sampling technique was used to select samples. The expected number of patient flow to diabetes clinic in two days of the week Monday and Tuesday was 170 per week. The total expected data collection period was 1 month and 2 weeks, andthe average number of patients expected to visit the clinic during this period were 1020. Dividing this expected number by total sample size gave sampling fraction K = 1020/371 = 2.7, thus every other patient was planned to be interviewed in order of arrival. However, since the weekly average number of patients who visited the clinic during this period was less than 170 due to COVID-19 pandemic the data collection period was extended for additional one month and two weeks to get the calculated sample size while keeping the sampling fraction as planned.

### Data collection tool and procedure

Structured questionnaire was used to conduct face-to-face interview of study subjects. The questionnaire was prepared in English, translated into Amharic and Afan Oromo, and then translated back to English to check its consistency. The Amharic and Afan Oromo versions were used for data collection after pretesting was done on 10% (37 DM patients) of the sample size attending Jimma higher 2-healthcenter. The questionnaire was developed by adapting various relevant tools from previous studies and modification was done after interviewing health care providers and nutrition researchers. Two multilingual nurses with previous experience in a similar study did the data collection and one public health officer supervisedthe data collection.

### Measurement and operational definition

Expanded Version of the Summary of Diabetes Self-Care Activities (SDSCA) adapted for Ethiopian context was used to assess self-care behaviors [17].The adaptation included changes in some foodstuffs and terminologies used in the original version to fit Ethiopian context. The SDSCA contains a set of items that measures frequency of various self-care activities in the last 7 days for each domain including dietary practice, foot-care, physical exercise,SMBG and taking medication. It is presented in terms of mean days for each domain. Internal consistency was checked during pre-test and was found Cronbach alpha of 0.8, 0.75, 0.83, and 0.76 for the diet, exercise, self-monitoring of blood glucose (SMBG), and foot care respectively. Respondents are regarded as having good dietary practice if he/she strictly follows dietary regimen ≥3 days per week other wise it is poor [18].

The modified Diabetes Knowledge Test (DKT) adapted for Ethiopian context was used to measure general diabetes knowledge including self-care [17]. DKT is a23-item multiple-choice questionnaire designed to assess knowledge about diabetes and self-care activities. It has originally 23 questions, 14 for both type of DM patients and 9 for those who took insulin only. Omitting the latter nine questions, 14 questions were selected for this research. Pre-testing has yield Cronbach alpha of 0.74.

Dietary practice perception was measured by using questionnaire developed by the investigator after reviewing different questionnaires that have been used in previously published researches [19, 20] and conducting interview with patients, health professionals, and public health experts to ensure its face validity. The questionnaire included 25 questions for all HBM constructs perceived susceptibility, severity, benefit, barrier and self-efficacy as well as cues to action. During pretesting internal consistency was checked and Cronbach alpha was 0.83, 0.73, 0.78, 0.85, 0.75, and 0.74 for the constructs perceived susceptibility, severity, benefit, barrier, self-efficacy and cues to action respectively.Perception questions was measured with 5-point Likert scale, 1-strongly disagree 2-disagree 3-neutral 4-agree and 5-strongly agree. Cues to action was measured using Yes/No response. Regarding the interpretation of scoring, higher scores indicates a good level of perception except for barrier which indicate bad perception [20].

Social support was measured using Multidimensional Scale of Perceived Social Support (MSPSS)questionnaire [21]. It contains 12 items rated on a 5-point Likert type scale. Pre-testing has yieldedCronbach alpha of 0.76. After adding up individual item score it was treated as a continuous variable for analysis. Higher scores indicate a good level of social support.

The other variable diabetes distress was measured by Diabetes Distress Scale(DDS) [22]. The scale has internal consistency with Cronbach alpha of 0.84 during pre-testing. The possible answer range from one to six (1 = not a problem 2 = slightly a problem 3 = a moderate problem 4 = somewhat a serious problem 5 = serious problem 6 = A very serious problem). Mean score was computed and treated as a continuous variable for analysis. Higher scores indicate a high level of distress.

### Study variables

#### Dependent variable

Dietary self-care practice.

#### Independent variable

Socio-demographic; -Age, sex, place of residence, marital status, educational status, occupation, average monthly income, family size, and availability of fruit and vegetable in a nearby market.

Clinical characteristic; -Type of diabetes, time since diagnosis of DM, types of treatment, comorbidity, family history of diabetes, attending diabetic education, membership in diabetes association, and access to self-monitoring of blood glucose(glucometer).

Psychosocial variables; -Diabetic knowledge, diabetes health belief i.e. Perceived susceptibility, severity of Diabetes complications, perceived benefits, Perceived barrier, cues to action, and self-efficacy. Family and social support as well as diabetes distress.

#### Association of variables

The association of independent variables to predict dietary self-care was assessed by two blocks, diabetic health belief block (perceived susceptibility, severity, benefit, barrier, self-efficacy, and cues to action) and modifying factor block (socio-demographic and socio-psychological variables). Diabetic health belief has direct effect on dietary practice, whereas modifying factors affect dietary practice through diabetic health belief constructs. In fact, some of modifying factors have additional direct path to dietary practice.

### Data processing and statistical analysis

Data entry was performed using Epi data version 3.1; then transported to IBM SPSS software and data cleaning, coding and recoding as well as checking for missing value was done. Before the final regression measurement model, with exploratory factor analysis, was done for constructs measuring perception, knowledge, social support, and diabetes distress questions. Using eigenvalue of 1 for extraction and varimax rotation 5 components were extracted. Perceived susceptibility and severity questions loading four and two items respectively. Two items loaded on perceived benefit of dietary self-care, four items on perceived barrierand four items on perceived self-efficacy towards dietary self-care practice. Total variance explained using those items was 72.79%. Similar procedures were followed for knowledge, social support and diabetes distress variables. Values for continuous quantitative data were presented using descriptive statistics. Percentage with frequency tables was used for categorical data. Tables and graphs were used to present data as required. A path analysis model with maximum likelihood estimation was fitted using STATA version 14 statistical software packageto test the hypothesized structural relationships. Model fitness was evaluated using absolute measures like Chi-Square statistic, and Root Mean Square Error of Approximation (RMSEA). Indices such as Tucker-Lewis Index (TLI), Comparative Fit Index (CFI), and SRMR were also used to test model fitness [23].

### Data quality control

Quality of data was assured through careful translation and back translation of questionnaire by independent translators. A two-day training on instrument, about data collection and supervision was given for data collectors and supervisor by principal investigator. A week before the actual data collection pretesting of the tool was done on 37 individuals (10%) of sample size in Jimma higher 2-healthcenter. Ambiguity in questions and clarity was discussed with data collectors every day during the pre-testing. Reliability of knowledge, social support, diabetes distress, and perception questions were checked for internal consistency by calculating Cronbach alpha values as reported above. Data were checked for completeness in the hospital during actual data collectionand on daily basis before entry.

### Ethical considerations

The study was approved by the ethical Committee of Jimma University. Before the start of each interview, verbal consent was asked from each respondents after an information session detailing the study, voluntary participation, and withdrawal from the study. Confidentiality was maintained by not writing respondents name and using the data only for research purpose. Precautions for COVID-19 were in place, data collection was made keeping the recommended physical distance as well as masks and sanitizers were provided for data collectors.

## Results

### Participants characteristics

#### Socio-demographic characteristics

From the 371 individuals planned to be interviewed 354 individuals responded which yields about 95.4% response rate. Of those 354 patients, 52% were male. The mean ± SD age of respondent is 41.4±13.7years. Regarding educational status of respondents 49(13.8%) respondent can’t read and write, while 193(54.5%) participants had secondary and above schooling. Around 23%of participants are unemployed while the rest were employed including self-employed. The median ± IQR of average family monthly income was 3600±2700 Ethiopian birr. 70.3% of individuals have fruit and vegetables available in their nearby market (see Table 1).

**Table 1:** Socio-demographic characteristics of diabetic patients in JUMC, 2020.

Variable category	Frequency	Percentage
**Sex**		
Male	184	52.0%
Female	170	48.0%
**Age**		
18–35	136	38.4%
36–50	126	35.6%
51–65	77	21.8%
>65	15	4.20%
**Residence**		
Rural	35	9.90%
Urban	319	90.1%
**Marital status**		
Currently single	158	44.6%
Currently married	196	55.4%
**Educational status**		
Primary& below schooling	161	45.5%
Secondary and above schooling	193	54.5%
**Occupation**		
Unemployed	81	22.9%
Government employee	73	20.6%
NGO employee	40	11.3%
Self-employee	160	45.2%
**Monthly income(Eth Birr.)**		
< = 3500	175	49.4%
>3500	179	50.6%
**Family size**		
<4	101	28.5%
> = 4	253	71.5%
**Availability of fruit and vegetable**		
Yes	249	70.3%
No	105	29.7%

#### Clinical characteristics

From the total participants in the study 75(21.2%) and 145(41%) participants are Type 1 and Type 2 DM patients respectively. mean± SD of disease duration since diagnosis is 4.59±2.7 year. About thirty-five percent of respondents were diagnosed for at least one diabetes complication such as Cardio Vascular Disease (CVD), renal or nerve disorder. About 272(76.8%) respondentsparticipate in diabetic education at least some times (see Table 2).

**Table 2:** Clinical and relevant characteristics of diabetic patients in JUMC, 2020.

Variables	Frequency	Percentage
**Type of DM**		
Type 1	75	21.2%
Type 2	145	41.0%
Don’t know	134	37.9%
**Type of medication**		
Injectable	103	29.1%
Oral	161	45.5%
Both	90	25.4%
**Duration since diagnosis**		
< = 5 year	229	64.7%
>5 year	125	35.3%
**Presence of diagnosed complication**		
Yes	130	36.7%
No	224	63.3%
**Follow diabetes education**		
Never	82	23.2%
Yes, sometimes	238	67.2%
Yes, usually	34	9.6%
**Member of diabetes association**		
Yes	33	9.3%
No	321	90.7%
**Family member with DM**		
Yes	93	26.3%
No	261	73.7%
**Presence of glucometer at home**		
Yes	119	33.6%
No	235	66.4%
**Alcohol drinking**		
Drinker	103	29.1%
Non-drinker	251	70.9%
**Smoking status**		
Smoker	37	10.5%
Non-smoker	280	79.1%
Ex-smoker	37	10.5%
**Khat chewing**		
Yes	146	41.2%
No	208	58.8%

#### Psychosocial characteristics

Concerning knowledge about general Diabetes Mellitus as well as self-care practices, the mean ± SD is 7.26±3.1. Regarding diabetes distress scale (DDS) result, meanscore ranges from 1.5 to 5.1 with overall mean ± SD score of 2.8±0.7. Though majority (57.6%) of respondents have mild diabetes distress of lower clinical significance, however a significant proportion (42.4%) of respondents scored 3 and above that contained them into level of distress worthy of clinical attention. Social support also ranges from minimum score of 22 to maximum score of 59 with 151(42.7%)of participant scoring above the mean value of 43.9.

Health belief model constructs were measured and the five constructs developed as explained above. The mean ± SD score is 15.4 ± 3.6 and 8.5±1.4 for perceived susceptibility and severity constructs respectively. For analysis purposes susceptibility and severity constructs summed up to create new variable called perceived threat. With regard to self-efficacy respondents scored for question ranging from four a minimum score, to twenty which is a maximum score with mean±SD value of 11.5±3.7 (see Table 3).

**Table 3:** Psychosocial characteristics of diabetic patients in JUMC, 2020.

Variables	Possible range	Observed range	Mean	SD
Knowledge	0–14	1–14	7.26	3.13
Diabetes distress***	1–6	1.53–5.06	2.85	0.68
Social support	12–60	22–59	43.95	7.59
HBM constructs				
Perceived threat^###^	6–30	9–30	23.96	4.22
Perceived susceptibility	4–20	5–20	15.43	3.57
Perceived severity	2–10	2–10	8.46	1.45
Perceived Dietary benefit	2–10	2–10	7.53	1.53
Perceived Dietary barrier	4–20	4–20	11.81	4.23
Self-efficacy	4–20	4–20	11.56	3.67
Cues to action	0–5	0–5	2.22	1.62

*** DDS is a mean score obtained by dividing sum of individual item score by 17.

### Susceptibility and severity scores summed up to create new threat variable for analysis.

### Level of self-care behavior

Regarding self-care behavior, Based on the cut-off of 3 and above mean days of practice, proportion of participant having good dietary practice is 42.4% (P = 42.4%, 95%CI = 37.2%-47.7%). The mean ± SD of overall self-care behavior is 3.2±1.0 day. Concerning individual self-care activities, the mean ± SD for dietary practice is 3.1±1.0. (see Table 4).

**Table 4:** General and domain diabetic self-care practice of diabetic patients in JUMC, 2020.

Self-care activities	Possible Range	Observed Range	Mean	SD
Overall self-care	0–7	1.2–5.7	3.2	1.0
Specific activities				
Dietary activities	0–7	1.2–5.6	3.1	1.0
Medication intake	0–7	1–7	6.1	1.3
Self-blood glucose monitoring	0–7	0–7	1.1	1.7
Physical exercise	0–7	0–7	3.1	1.6
Foot care	0–7	0–7	3.8	1.8

### Predictors of dietary practice

#### Model fit information

Model fitness was tested using absolute as well as comparative fit tests. Though χ^2^ value was significant, however its value when divided by degree of freedom gave<5. In addition, RMSEA value is 0.054 and 90%CI upper bound is 0.08 which is less than one. CFI and TLI value is also above the recommended >0.9 value. SRMR is also bellow the recommended value of 0.08. modifications from original model was done by adding covariance term between self-efficacy and perceived benefit constructs, and adding some direct paths from modifying factors to dietary practice which were not initially planned. (see Table 5).

**Table 5:** A path model fit indices of dietary self-care practice, health belief model.

Indices		Value
χ^2^ statistics (degree of freedom)		42.5 (21)
	*P* >χ^2^	0.004
	χ^2^/df	2.0
RMSEA (90% CI)		0.05 (0.03, 0.08)
	*P* ≤0.05	0.34
CFI		0.98
TLI		0.933
SRMR		0.02

CFI: Comparative Fit Index; RMSEA: Root Mean Square Error of Approximation; TLI: Tucker Lewis Index; SRMR: Standardized Root Mean squared Residual.

#### Bivariate association of variables

Pairwise correlation association of variables was done to see association of variables with general self-care and dietary practice. Mean dietary self-care has weak correlation with socio-demographic variables except for educational status (r = 0.3), and monthly income (r = 0.3) that has moderate significant correlation. Age, place of residence, marital status and family size are found to have non-significant correlation with dietary practice. Regarding clinical characteristics while having family member with diabetes has no significant correlation; duration since diagnosis has showed weak significant correlation with r = 0.3. From psycho-social variables perceived threat (r = 0.5), perceived benefit (0.4), perceived barrier (r = -0.5), self-efficacy (r = 0.6), and cues to action (r = 0.5) have showed significant strong correlation. In addition, knowledge, social support and diabetes distress is correlated with dietary self-care with moderate and above strength. All HBM constructs have significant correlations to each other.

#### Predictors of dietary practice

Age, sex, educational status, monthly income, availability of fruit and vegetable, duration since diagnosis, knowledge, social support, and diabetes distress, along with all HBM constructs are entered in to the path model to find independent predictors of dietary self-care practice according to previously specified path. Some variables that were not in original HBM, like duration since diagnosis and fruit availability, were added after examining their correlation with dietary self-care.

Patients perception of threat of diabetes significantly predicted dietary self-care practice (Unstandardized Total Effect±Standard Error (UTE±SE) = 0.02±0.01). Perceptions regarding barrier to dietary self-care (UTE±SE = -0.08±0.01) is found to predict dietary self-care practicesignificantly. Self-efficacy is the strongest construct among diabetic belief that has highest standardized direct effect and the second construct with standardized total effect after cues to action (UTE±SE = 0.10±0.01). Cues to action has indirect significant effect on dietary self-care practice through perceived barrier construct of HBM (UTE±SE = 0.17±0.05).

Other variables which significantly predict dietary self-care are knowledge (UTE±SE = 0.22±0.03), social support (UTE±SE = 0.02±0.01), diabetes distress (UTE±SE = -0.03±0.004), and duration since diagnosis (UTE±SE = 0.05±0.01). Male sex as well as secondary and above schooling level are also significantly associated variable with higher dietary self-care practice with UTE±SE of 0.10±0.04 and 0.27±0.06 respectively. Age, and average monthly income are also variables with significant total effect on dietary self-care (see Table 6).

**Table 6:** Total, direct and indirect effects of dietary self-care practice, JUMC 2020.

Variables	Unstandardized effects / Path coefficient(SE)		
	Direct Effect(SE)	Indirect Effect(SE)	Total Effect(SE)
**HBM constructs**			
Perceived threat	0.02(0.01)**	**NP**	0.02(0.01)**
Perceived benefit	0.04(0.03)	NP	0.04(0.03)
Perceived barrier	-0.08(0.01)**	NP	-0.08(0.01)**
Self-efficacy	0.10(0.01)**	NP	0.10(0.01)**
Cues to action	0.07(0.05)	0.10(0.02)**	0.17(0.05)**
**Modifying factors**			
Knowledge	0.08(0.03)*	0.09(0.02)**	0.22(0.03)**
Social support	NP	0.02(0.005)**	0.02(0.01)**
Diabetes distress	NP	-0.03(0.004)**	-0.03(0.004)**
Duration since diagnosis	NP	0.05(0.01)**	0.05(0.01)**
Income	NP	0.20(0.05)**	0.20(0.05)**
Age	NP	0.007(0.002)**	0.007(0.002)**
Sex	NP	0.10(0.04)*	0.10(0.04)*
Educational status	NP	0.27(0.06)**	0.27(0.06)**
Fruit and vegetable availability	NP	0.06(0.04)	0.06(0.04)
**Covariance**		**Coef.(95%CI)**	**P-value**
e. perceived benefit, e. self-efficacy		**1.15(0.77–1.53)**	**<0.001**

NP:—No Path.

* Association is significant at the 0.01 level (2-tailed).

** Association is significant at the 0.01 level (2-tailed).

### Effect decomposition of important variables

While the direct effect is insignificant cues to action has shown significant indirect effect on dietary self-care practice through perceived barrier (Unstandardized Direct Effect(UDE±SE = -1.14±0.26)). In addition to its significant direct effect indirect effect of knowledge is significant through perceived threat (UDE±SE = 1.09±0.14), perceived barrier (UDE±SE = -0.63±0.17), and self-efficacy (UDE±SE = 0.35±0.12); however, path through diabetes distress is non-significant. Social support affects dietary practice via perceived threat (UDE±SE = 0.23±0.03), self-efficacy perception (UDE±SE = 0.13±0.003), and diabetes distress (UDE±SE = -0.33±0.05). Perceived threat (UDE±SE = -0.08±0.03), perceived barrier (UDE±SE = 0.21±0.04) and self-efficacy (UDE±SE = -0.10±0.02) are paths through which distress affect dietary self-care.

**Fig 1:**
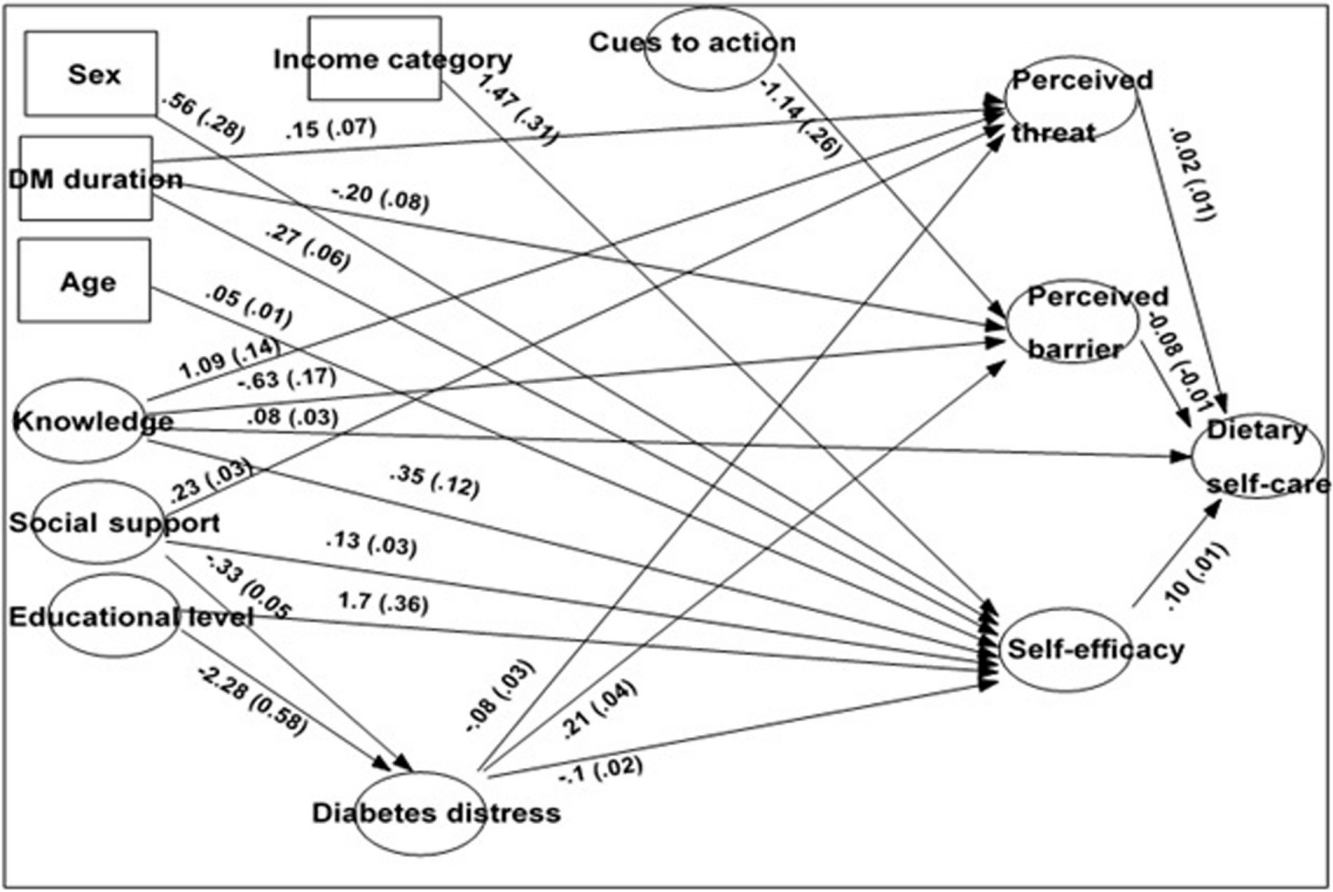
Unstandardized path coefficient (Standard error) of significant paths predicting dietary self-care practice among DM patients in JUMC, 2020.

Indirect effect of age is significant through self-efficacy (UDE±SE = 0.05±0.01); where as its effect through perceived threat, perceived benefit and barriers are non-significant. Being male increase dietary self-care behavior through self-efficacy (UDE±SE = 0.56±0.28); whereas average monthly income of >3500 birr showed statistically significant indirect effect via increasing self-efficacy(UDE±SE = 1.47±0.31). Secondary and above schooling is also found to affect dietary practice through increasing one’sself-efficacy perception (UDE±SE = 1.70±0.36), and reducing diabetes distress (UDE±SE = -2.28±0.58). Duration since diagnosis appears to affect dietary self-care indirectly via reducing perceived barrier (UDE±SE = -0.20±0.08) whileincreasing perceived threat (UDE±SE = 0.15±0.07), and self-efficacy (UDE±SE = 0.27±0.06). (Table 7 and Fig 1 for detail).

**Table 7:** Decomposition of modifying factors indirect effects on dietary practice, JUMC 2020.

Variables	Effect/ Path coefficient		
	Standardized	Unstandardized(SE)	P-value
**Cues to action affects via**			
Perceived threat	0.04	0.21(0.22)	0.352
Perceived barrier	**-0.21**	**-1.14(0.26)**	**<0.001**
**Knowledge affects via**			
Perceived threat	**0.33**	**1.09(0.14)**	**<0.001**
Perceived benefit	0.19	0.21(0.06)	<0.001
Perceived barrier	**-0.19**	**-0.63(0.17)**	**<0.001**
Self-efficacy	**0.13**	**0.35(0.12)**	**0.003**
Diabetes distress	-0.06	-0.29(0.22)	0.197
**Social support affects via**			
Perceived threat	**0.33**	**0.23(0.03)**	**<0.001**
Perceived benefit	0.33	0.08(0.01)	<0.001
Perceived barrier	0.10	0.07(0.04)	0.074
Self-efficacy	**0.21**	**0.13(0.03)**	**<0.001**
Diabetes distress	**-0.35**	**-0.33(0.05)**	**<0.001**
**Diabetes distress**			
Perceived threat	**-0.11**	**-0.08(0.03)**	**0.016**
Perceived benefit	-0.15	-0.04(0.01)	0.004
Perceived barrier	**0.29**	**0.21(0.04)**	**<0.001**
Self-efficacy	**-0.17**	**-0.10(0.02)**	**<0.001**
**Age**			
Perceived threat	0.08	0.02(0.01)	0.095
Perceived benefit	0.06	0.01(0.005)	0.243
Perceived barrier	-0.04	-0.01(0.01)	0.431
Self-efficacy	**0.18**	**0.05(0.01)**	**<0.001**
**Sex**			
Perceived barrier	-0.06	-0.52(0.41)	0.199
Self-efficacy	**0.08**	**0.56(0.28)**	**0.044**
**Educational status**			
Perceived threat	0.05	0.43(0.43)	0.319
Perceived benefit	-0.04	-0.12(0.17)	0.476
Perceived barrier	-0.03	-0.25(0.50)	0.613
Self-efficacy	**0.23**	**1.70(0.36)**	**<0.001**
Diabetes distress	**-0.20**	**-2.28(0.58)**	**<0.001**
**Income**			
Perceived threat	0.03	0.25(0.37)	0.506
Perceived benefit	-0.02	-0.06(0.15)	0.670
Perceived barrier	-0.07	-0.56(0.43)	0.197
Self-efficacy	**0.20**	**1.47(0.31)**	**<0.001**
**Duration since diagnosis**			
Perceived threat	**0.09**	**0.15(0.07)**	**0.032**
Perceived barrier	**-0.13**	**-0.20(0.08)**	**0.013**
Self-efficacy	**0.19**	**0.27(0.06)**	**<0.001**

The number in the above figure indicates the unit change in perception for arrows towards perception constructs and the change in the number of days for arrows towards dietary self-care.

## Discussion

In this study, mean (SD) of general self-care was 3.16(0.98) and that of dietary self-care was 3.14(1.01) resulting good self-care proportion of 48% and 42.4% for general self-care and dietary practice respectively. This study reported proportion of good dietary practice lower than results found from Nepal study [24], and a study done in Tribhuwan University [25], while closer result was recorded in Bangladesh [26]. This could be due to the fact that in these countries socio-economical and health care facilities are somehow more improved than Jimma. It is also lower than findings from Nigeria [27] and Botswana [28] in which 2/3^rd^ of patients adhered to dietary self-care. While two studies done AddisAbaba [16] and study from Arbaminch [29] revealed higher result of good dietary practice than here, however current study result is higher than studies in Debre-tabor [30] and Felegehiwot hospitals [31]. This difference might be attributed to the method of dietary practice assessment tool since most of them used Moriski adherence tool, PDAQ, and others where as SDSCA was used in this study.

Individuals with high threat perceptions have more practice days than those with lower perception. This is similar with the study in Harar which showed patients with high severity perceptions more likely practice self-care than those with lower perception [20]. This is also in accordance with the basic assumptions of health belief model that individual’s likelihood of engaging in healthy behavior is higher as their perceived susceptibility and severity gets higher. This is because healthy behaviors are practiced to prevent complications and this complication should be perceived or one needs to think that he or she is at risk of harboring illness conditions before acting to prevent them.

Participant who had higher unit barrier perception are found to have less days of dietary practice than participants with lower perceived barrier to dietary self-care. Similar result was found from a study in Yekatit hospital [32] that patients who think about cost of good dietary practice are twice more likely engage in poor dietary practice. Studies in Harari [33] and Tigray [34] have also showed that patients with high barriers have less odds of practicing good self-care in general than patients with low barrier perceptions. As Rosenstock and others have said this might be due to the reason that a kind of cost-benefit analysis is thought to occur wherein the patients prefer action’s effectiveness over feelings that it may be expensive, unpleasant (e.g., painful, difficult, upsetting), inconvenient, time-consuming, and the like [35].

Regarding self-efficacy perception, it is found not only significantly effecting dietary perception but also it is the strongest predictor among constructs predicting dietary practice. This is in line with result found in Malaysia study [22] as well as Ardakan city, Iran that Self-efficacy was the strongest predictor of self-care behavior [36]. This is also in accordance with core assumptions of HBM that individuals should believe that they are self-efficacious in successfully executing the behavior required to produce the outcomes [35].

In this study as patients receive more cues to action, they tend to have more mean days of dietary practice. This finding is in line with Nepal [8] study as well as core assumptions of HBM that is as individuals have more cues to action they are less likely to forget advices, since readiness to take action could only be potentiated by cues to inaugurate it [35].

In this study, higher knowledge was found to increase dietary practice significantly. otherslike west Shoa [37], Benishangul [38] and Nekemt studies [39] also reported an association of dietary knowledge and dietary practice. The reason might be that patients having more knowledge regarding diabetes had less barrier and more self-efficacy perception that could lead them to higher practice score.

Those with higher diabetes distress were found to practice lesser days than those with lower distress score. this finding is in line with result from study in Boston [40] and other systematic review study [41] which reported negative association of diabetes distress and dietary practice. Thismight be due to the reason that diabetes distress reduce self-confidence while increasing barrier perception to perform recommended dietary practice. Additionally, depressed mood could inhibit adherence by decreasing the desire to seek treatment, thus making mediator of the link between depression and poor diabetes outcomes.

Social support is another significantly associated factor, which increases dietary self-care, as it gets higher and higher. This finding is similar with study in Nepal [8], Iran [36], and Felegehiwot [31] hospitals which found dietary practice is associated with social support. As shown in the path, this might be due to the reason that since social support helps in coping self-care fatigue, as social support increases diabetes distress decreases, whereas self-efficacy increase thereby improving patients dietary practice indirectly.

Increased age as well as duration since diagnosis were also related with good dietary practice in this study. This finding is in contrast with Nepal study [24]. This could be because of experiencing symptoms of both acute as well as chronic complication in both factors and exposure for repeated counseling and health educations in longer duration might act as cues to act healthily.

In this study, females are found to have low dietary practice by decreasing self-efficacy. This is similar with Nepal study [24]. This might be due to the reason that in developing countries females have less access to education and income generating activities, which both have important relation with once self-confidence of engaging in good dietary activities.

Attaining secondary and above schooling also increased dietary practice of patients in this study. This finding is similar with studies in Ethiopia [16, 30, 42] and other African countries [27, 28] that found higher educational level is associated with good dietary practice. As shown in the path, this might be due to the reason that educated ones are capable of being informed and self-efficacious in interpreting as well as performing recommendations.

Those with >3500 Birr monthly income is associated with good dietary practice than those with lower monthly income. This is consistent with Debre-tabor and in contrast from Tikur Anbessa study that found high monthly income is associated with good dietary practice [30, 42]. The reason might be having high income makes patients more concerned to their health status as well as increase self-confidence to perform this behavior to reduce their likelihood of complication.

The above results and discussion all culminates to and implies that dietary practice is a very important however, very under practiced self-care domain. In addition, health belief model especially self-efficacy and cues to action constructs can best explain dietary practice variation there by used to design appropriate health promotion intervention for improved dietary practice.

### Limitation

First the study is cross sectional and it is institution based. As institution based studies don’t give full picture and cross sectional studies temporality issue is a concern cautions should be taken when interpreting and using results of this study. Second practice level was measured using self-report, that desirability may arise and trustworthiness could not be ensured.

## Conclusion

In JUSH less than half of patients have good dietary and general self-care practice. Self-efficacy being the strongest; cues to action, perceived threat, and perceived barrier constructs of health belief model are found to predict dietary self-care. Knowledge, social support, diabetes distress and other socio-demographic characteristics modify patients’ perception thereby exerting significant influence on their dietary self-care practice. Therefore, individualized, perception based counseling, and patient centered problem solving care is needed to address thesevast socio-psychological factors in order to improve dietary as well as general self-care practice.

## Supporting information

S1 File English version questionnaire. (DOCX)

S2 File Amharic version questionnaire. (DOCX)

S3 File Data set of the study. (XLS)

## Abbreviations

ADA: American Diabetes Association
BMI: Body Mass Index
CFI: Comparative Fit Index
DKT: Diabetes Knowledge Test
DM: Diabetes Mellitus
HBM: Health Belief Model
IDF: International Diabetes Federation
JUMC: Jimma University Medical Centre
SDSCA: Summary of Diabetes Self-Care Activities
SE: Standard Error
SEM: Structural Equation modeling
STE: Standardized Total Effect
UDE: Unstandardized Direct Effect
