# Supplementary material for: Dietary self-care and associated factors among diabetic patients in Jimma University Medical Centre, South West Ethiopia; A path analysis
Source: PLoS One. 2022 Aug 24;17(8):e0273074. doi: 10.1371/journal.pone.0273074 (PMC9401131; doi:10.1371/journal.pone.0273074)
Supplement: S1 File — (DOCX) [file pone.0273074.s001.docx]

**questionnaire**

Jimma University, Institute of Health Science

This questionnaire assesses Socio-demographic characteristic, diabetes knowledge, self-care practice, social-support, diabetes distress, and diabetes perception among diabetic patients in JUMC. Jimma, Southwest Ethiopia, 2020

**Consent form**

**Good morning/ good afternoon!**

My name is ________________. I am here to collect data on behalf of Musa Jemal 2^nd^ year masters of Science in Human Nutrition student. He is conducting a research in diabetic follow up clinic of Jimma University Medical Center to assess dietary self-care behaviors and Associated factors in diabetic patients. You have been chosen to participate in this study by chance and you will help me by answering the questions. I only ask questions because the study doesn’t need to do any experiments or apply any invasive procedure up on you except your will to spent some time for interview.

I assure you that whatever answers you give it is kept strictly secret. I do not need your name and address. I also inform you that you have the right to withdraw from the study or stop the interview at any time if there is any discomfort before completing the study.

The interview takes approximately 20-25 minutes. if you have any questions you can ask.

Thank you very much!

Are you willing to participate in this study?

Yes_______________ No______________

If yes, go to the next page, completing the questionnaire

**Questionnaire**

**Part I Socio-demographic characteristics questions**

| No | Question | Response |
| --- | --- | --- |
| 101 | Age | __________ Years |
| 102 | Sex | 1.male 2.female |
| 103 | Residence | 1.Urban 2.Rural |
| 104 | Marital status | 1.single  2.married  3.divorced  4.separated  5.widowed |
| 105 | Educational level | 1.can’t read and write  2.can read and write  3.primary school  4.secondary and preparatory school  5.college /university/ technique |
| 106 | Occupation | 1.unemployeed  2.government employee  3.NGO employee  4.self-employee |
| 107 | Average monthly income | ______________birr |
| 108 | Family size | ______________ |
| 109 | Is there fruit and vegetable in a nearby market around your home ? | 1.Yes 2.No |
|  |  |  |
|  | **Part II Clinical characteristics** |  |
|  |  |  |
| 201 | Type of DM (self) | 1.Type 1  2.Type 2  3.i don’t Know |
| 202 | Duration since diagnosis (in month if <1 yr) | _______________yr/month |
| 203 | Type of treatment | 1.injectin  2.tablet  3.both  4.no medication |
| 204 | Comorbidity (from record/registration) | 1.yes  2.no |
| 205 | Do you attend diabetic education | 1.no never  2.yes sometimes  3.yes regularly |
| 206 | Are you a member of diabetic association | 1.yes  2.no |
| 207 | Are there any one who is diabetic patient in your family | 1.yes  2.no |
| 208 | Do you have your own glucometer at home | 1.yes  2.no |

**Part III: Summary of diabetes self-care activities questionnaires:**

The questions below ask you about your diabetes self-care activities during the past 7 days. If you were sick during the past 7 days, please think back to the 7 days that you were not sick.

| **S.No** | **Questions** | **Response in number of days** | | | | | | | |
| --- | --- | --- | --- | --- | --- | --- | --- | --- | --- |
|  | **Diet** | **0** | **1** | **2** | **3** | **4** | **5** | **6** | **7** |
| 301 | How many of the last SEVEN DAYS have you prepared a healthful eating plan? |  |  |  |  |  |  |  |  |
| 302 | On average over the past month, how many DAYS PER WEEK have you followed your eating plan? |  |  |  |  |  |  |  |  |
| 303 | On how many of the last SEVEN DAYS did you eat five or more servings of fruits and vegetables? |  |  |  |  |  |  |  |  |
| 304 | On how many of the last SEVEN DAYS did you eat high fat foods Such as red meat or full fat dairy products? |  |  |  |  |  |  |  |  |
| 305 | On how many of the last SEVEN DAYS did you space carbohydrates evenly through the day? |  |  |  |  |  |  |  |  |
|  | **Physical Activity** |  |  |  |  |  |  |  |  |
| 306 | On how many of the last SEVEN DAYS did you participate in at least 30 minutes of physical activity?(total minutes of continuous activity, including walking ) |  |  |  |  |  |  |  |  |
| 307 | On how many of the last SEVEN DAYS did you participate in a specific exercise session (such as swimming, walking, biking) other than what you do around the house or as part of your work? |  |  |  |  |  |  |  |  |
|  | **Blood Sugar Testing** |  |  |  |  |  |  |  |  |
| 308 | On how many of the last SEVEN DAYS did you test your blood sugar? |  |  |  |  |  |  |  |  |
| 309 | On how many of the last SEVEN DAYS did you test your blood sugar the number of times recommended by your health care provider? |  |  |  |  |  |  |  |  |
|  | **Foot care** |  |  |  |  |  |  |  |  |
| 310 | On how many of the last SEVEN DAYS did you check your feet? |  |  |  |  |  |  |  |  |
| 311 | On how many of the last SEVEN DAYS did you inspect the inside of your shoes? |  |  |  |  |  |  |  |  |
|  | **Medication** |  |  |  |  |  |  |  |  |
| 312 | On how many of the last SEVEN DAYS did you take your recommended diabetes medication? |  |  |  |  |  |  |  |  |

**Part IV: diabetic health belief question**

1 = strongly disagree 2 = disagree 3= Neutral 4= agree 5= Strongly disagree

| **S.No** | **Perceived susceptibility** | Strongly disagree | Disagree | Neutral | Agree | Strongly agree |
| --- | --- | --- | --- | --- | --- | --- |
| PT01 | As a diabetic patient, I am at risk of getting diseases like (kidney , heart and hypertension) |  |  |  |  |  |
| PT02 | As a diabetic patient, it is possible through process that I will get diseases like (kidney, heart, hypertension) |  |  |  |  |  |
| PT03 | As a diabetic patient, I have a chance of getting foot ulcer/gangrene |  |  |  |  |  |
| PT04 | As a diabetic patient, I have a chance of experiencing hypoglycemia |  |  |  |  |  |
|  | **Perceived severity** |  |  |  |  |  |
| PT05 | Experiencing diseases like kidney, heart and hypertension is a serious problem to diabetic patient. |  |  |  |  |  |
| PT06 | Getting diseases like kidney, heart and hypertension is life threating to diabetic patient |  |  |  |  |  |
| PT07 | Getting foot ulcer/gangrene leads diabetic patients to loss of body parts. |  |  |  |  |  |
| PT08 | Experiencing hypoglycemia can lead diabetic patient to sudden deaths |  |  |  |  |  |
| **Perceived benefit** | | | | | | |
| PB01 | The diabetes diet make me feel better |  |  |  |  |  |
| PB02 | If I change my eating habit it will probably help me |  |  |  |  |  |
| PB03 | I believe that my diet will control my diabetes |  |  |  |  |  |
| **Perceived barrier** | | | | | | |
| PB04 | The foods on the diabetic diet taste horrible |  |  |  |  |  |
| PB05 | It has been difficult what the doctor told (prescribed) for me about diet |  |  |  |  |  |
| PB06 | I cannot understand what my doctor told me about my diet. |  |  |  |  |  |
| PB07 | following the recommended diet interferes with my normal daily activities |  |  |  |  |  |
| **Self-efficacy** | | | | | | |
| SE01 | As a diabetic patient, it is easy for me to consume foods [like vegetables, fruits, low salt etc.] to prevent risks from diseases like kidney, heart and hypertension |  |  |  |  |  |
| SE02 | As a diabetic patient I have confidence to consume foods [like vegetables, fruits, low salt etc.] to prevent risks from diseases like kidney, heart and hypertension |  |  |  |  |  |
| SE03 | I am confident that I can stay on my meal plan when people around me don't know that I have diabetes. |  |  |  |  |  |
| SE04 | I am confident that I can eat meals at the same time every day. |  |  |  |  |  |
| SE05 | I am confident that I can avoid overeating or missing meals when I feel happy or angry. |  |  |  |  |  |

**Cues to action questions**

|  |  | 1. Yes | 2. No |
| --- | --- | --- | --- |
| CA01 | Do you have a family member with diabetes complication? |  |  |
| CA02 | Have you ever seen /heard about a person who follow recommended self-care practice in last one month |  |  |
| CA03 | Have you ever seen /heard of person having diabetes complication in the last one month |  |  |
| CA04 | Have you ever heard though media /newspaper about follow recommended self-care practice during last one month? |  |  |
| CA05 | Have you ever received leaflets post cards or anything else from hospital which reminds you of diabetes |  |  |

**Part V: social support question**

Now I am going to ask you about social support that you receive from families, friends and significant others. Please follow statement carefully and check “√” your response to what degree you agree or dis agree with it.

| **S.No** | **Question** | **Response** | | | | |
| --- | --- | --- | --- | --- | --- | --- |
|  |  | Strongly disagree | Disagree | Neutral | Agree | Strongly agree |
|  | **Family** |  |  |  |  |  |
| SS01 | I get emotional help and support from my family |  |  |  |  |  |
| SS02 | My family really tries to help me |  |  |  |  |  |
| SS03 | My family is willing to help me make decision |  |  |  |  |  |
| SS04 | I can talk about my problem with my family |  |  |  |  |  |
|  | **Friends** |  |  |  |  |  |
| SS05 | My friends really try to help me |  |  |  |  |  |
| SS06 | I can count on my friends when things go wrong |  |  |  |  |  |
| SS07 | I can talk about my problem with my friends |  |  |  |  |  |
| SS08 | I have friends with whom I can share my joys and sorrows |  |  |  |  |  |
|  | **Significant others** |  |  |  |  |  |
| SS09 | There is special person in my life that cares about my feeling |  |  |  |  |  |
| SS10 | I have special person who is real source of comfort to me |  |  |  |  |  |
| SS11 | There is a special person who is around when I am in need |  |  |  |  |  |
| SS12 | There is a special person with whom I can share my joys and sorrows |  |  |  |  |  |

**Part VI: Diabetes and self-care Knowledge question**

Here are 14 question to measure Knowledge on diabetes and self-care please choose the correct answer after following both the question and answer attentively

| 601 | The diabetes diet is: | |
| --- | --- | --- |
|  | a. The way most Ethiopian people eat | b. A healthy diet for most people |
|  | c. Too high in carbohydrate for most people | d. Too high in protein for most people |
| 602 | Which of the following is highest in carbohydrate? | |
|  | a, Baked chicken | b. Ergo |
|  | c. Baked potato | d. Peanut butter |
| 603 | Which of the following is highest in fat? |  |
|  | a. Milk | b. Orange juice |
|  | c. Corn | d. Honey |
| 604 | Which of the following is a “sugar free food”? | |
|  | a. Any unsweetened food | b. Any dietetic food |
|  | c. Any food that says “sugar free” on label | d. Any food that has less calories |
| 605 | Fast blood sugar is a test that is a measure of your blood glucose level for the past: | |
|  | a. Day | b. Week |
|  | c. 6 weeks | d. 6 months |
| 606 | Which is the best method for testing blood glucose? | |
|  | a. Urine testing | b. Blood testing |
|  | c. Both are equally good |  |
| 607 | What effect does unsweetened fruit juice have on blood glucose? | |
|  | a. Lowers it | b. Raises it |
|  | c. Has no effect |  |
| 608 | Which should not be used to treat low blood glucose? | |
|  | a. 3 hard candies | b. 1/2 cup orange juice |
|  | c. 1cup soft drink | d.1cup milk |
| 609 | For a person in good control, what effect does exercise have on blood glucose? | |
|  | a. Lowers it | b. Raises it |
|  | c. Has no effect |  |
| 610 | Infection is likely to cause: | |
|  | a. An increase in blood glucose | b. Decrease in blood glucose |
|  | c. No change in blood glucose |  |
| 611 | The best way to take care of your feet is to: | |
|  | a. Look at and wash them each day | b. Massage them with alcohol each day |
|  | c. Soak them for one hour each day | d. Buy shoes a size larger than usual |
| 612 | Eating foods lower in fat decreases your risk for: | |
|  | a. Nerve disease | b. Kidney disease |
|  | c. Heart disease | d. Eye disease |
| 613 | Numbness and tingling may be symptoms of: | |
|  | a. Kidney disease | b. Nerve disease |
|  | c. Eye disease | d. liver disease |
| 614 | Which of the following is usually not associated with diabetes? | |
|  | a. Vision problems | b. Kidney problems |
|  | c. Nerve problems | d. Lung problems |

**Part VII Diabetes associated distress question**

Below are 17 questions regarding diabetes-associated distress. Follow the statements attentively and answer to what degree the statements apply to you as a problem then check “√” it on the response space.

1= Not a problem 2= slightly a problem 3= A moderate problem 4= somewhat a serious problem 5= Serious problem 6= A very serious problem

| S.No | Question | Response | | | | | |
| --- | --- | --- | --- | --- | --- | --- | --- |
|  |  | 1 | 2 | 3 | 4 | 5 | 6 |
| 701 | Feeling that diabetes is taking up too much my mental and physical energy |  |  |  |  |  |  |
| 702 | Feeling that my doctor doesn’t know enough about diabetes and diabetes care |  |  |  |  |  |  |
| 703 | Feeling angry, distressed or depressed when I think about living with diabetes |  |  |  |  |  |  |
| 704 | Feeing that my doctor doesn’t give me clear enough direction on how to manage diabetes |  |  |  |  |  |  |
| 705 | Feeling that I am not testing my blood sugar frequently enough |  |  |  |  |  |  |
| 706 | Feeling that I am often failing with my diabetes routine |  |  |  |  |  |  |
| 707 | Feeling that families or friends are not supporting enough of self-care effort (eg. Planning activities that conflict with my schedule, encouraging me to eat the ‘wrong’food |  |  |  |  |  |  |
| 708 | Feeling that diabetes controls my life |  |  |  |  |  |  |
| 709 | Feeling that my doctor doesn’t take my concern seriously enough |  |  |  |  |  |  |
| 710 | Not feeling confident in my day to day ability to manage diabetes |  |  |  |  |  |  |
| 711 | Feeling that I will end up with serious long term complication, no matter what I do |  |  |  |  |  |  |
| 712 | Feeling that I am not sticking enough to a good meal plan |  |  |  |  |  |  |
| 713 | Feeling that friends or family don’t appreciate how difficult living with diabetes can be |  |  |  |  |  |  |
| 714 | Feeling overwhelmed by the demand of living with diabetes |  |  |  |  |  |  |
| 715 | Feeling that I don’t have a doctor who I can see regularly enough about my diabetes |  |  |  |  |  |  |
| 716 | Not feeling motivated to keep up my diabetes self-management |  |  |  |  |  |  |
| 717 | Feeling that family or friends don’t give me the emotional support that I would like |  |  |  |  |  |  |

PartVIII behavioural questions

| S.No | Question | Response | Remark |
| --- | --- | --- | --- |
| D01 | Have you ever smoked? | 1.Yes  2.No | If no go to D05 |
| D02 | Do you currently smoke? | 1.Yes  2.No |  |
| D03 | If yes do you smoke daily? | 1.Yes  2.No |  |
| D04 | How many days do you smoke per week? | _______ |  |
| D05 | Did you chew Khat in the last 12 month? | 1.Yes  2.No | If No go to D07 |
| D06 | If yes how many days per week do you chew per week? | _________ |  |
| D07 | Have you ever drink an alcohol? | 1.Yes  2.No | If No leave the remaining |
| D08 | Did you drink alcohol in the last 12 months? | 1.Yes  2.No |  |
| D09 | If yes how many days per week do you drink at least one? | 1.Daily  2.5-6 days per week  3.1-4 days per week  4.1-3 days per month  5.Less than one day per month |  |
