# Supplementary material for: Dietary self-care and associated factors among diabetic patients in Jimma University Medical Centre, South West Ethiopia; A path analysis
Source: PLoS One. 2022 Aug 24;17(8):e0273074. doi: 10.1371/journal.pone.0273074 (PMC9401131; doi:10.1371/journal.pone.0273074)
Supplement: S2 File — (DOCX) [file pone.0273074.s002.docx]

**የአማርኛትርጉምየመጠየቂያፎርም**

ጅማ ዩኒቨርሲቲየህብረተሰብእናየህክምናሳይንስትምህርትኮሌጅበደቡብምዕራብጅማዞን፤በጅማ ዩኒቨርሲቲ ሆስፒታል ውስጥ ያሉ የስኳር ህመምተኞች የስኳር በሽታ ምልከታ እና ስለሚያደርጉትየግልእንክብካቤ፤ለማወቅየተዘጋጀየመጠይቅፎርምነው፡፡ ከመጠየቅበፊትየተዘጋጀየፍቃድጥያቄፎርም፡-

ሰላምታ

መግቢያ

ስሜ__________________ ይባላል፡፡እኔየመጣሁት በጅማዩኒቨርሲቲበሚገኘው በሙሳ ጀማል ቦታ ነው፡፡ይህመጠይቅበጅማዩኒቨርሲቲሆስፒታል በስኳርበሽታመከታተያክፍልውስጥህመምተኞችየስኳር በሽታ ምልከታ እና ስለሚያደርጉትየግልእንክብካቤ፤ለማወቅየሚደረግመጠይቅነው።እርሶየተመረጡትበአጋጣሚነው።በእርሶላይየሚደረግምንምአይነትምርመራእንደሌለተገንዝበውከእርሶየሚጠበቀውየተወሰነደቂቃለቃለምልልስተባባሪእንዲሆኑብቻነው፡፡

በመጠይቁላይየእርሶንስምወይምማንነትየሚገልፅማንኛውምነገርአይጠቀስም፤እንዲሁምእርሶለሚሰጡኝመረጃዎችሚስጥራዊነትለመጠበቅያመችዘንድመጠይቁእኔናእርሶባለንበትቦታብቻይከናወናል፡፡መጠይቁሚከናወነውበእርሶፍቃደኝነትብቻየሚሆንሲሆንበመጠይቁወቅትመመለስየማይፈልጉትንማንኛውምዐይነትጥያቄይለፈኝማለትይችላሉ፡፡በተጨማሪምበማንኛውምሰዓትጥያቄውንማስቀረት/መተውይችላሉ፡፡ሆኖምእርሶየሚሰጡትትክክለኛመረጃዎችለስኳርህክምናአገልግሎትመስተካከልናመሻሻልስላለባቸውነገሮችለማወቅስለሚረዳንከፍተኛጥቅምአለው፡፡

ቃለምልልሱከ20-25 ደቂቃይወስዳል፡፡

ጥያቄአለህ / አለሽ? መጠየቅትችላለህ

በጣምአመሰግናለሁ!!

በጥናቱላይለመሳተፍፍቃደኛኖዎት)

አዎበፊርማዎ ያረጋግጡልኝ______________________________________

አይደለሁም

መልስህአዎከሆነወደሚቀጥለውገፅበመሄድጥያቄዎውንይመልሱ፡፡

**ክፍል 1- የማህበራዊ ሁኔታን የተመለከቱ ጥያቄዎች**

| **ተ/ቁ** | **ጥያቄዎች** | **አማራጭ መልሶች** |
| --- | --- | --- |
| SD01 | እድሜ | ______ዓመት |
| SD02 | ፆታ | 1.ወንድ 2.ሴት |
| SD03 | መኖሪያ ቦታ | 1.ከተማ 2.ገጠር |
| SD04 | የጋብቻ ሁኔታ | 1. አላገባሁም  2. አግብቻለሁ  3. ተፋተናል  4. ተለያይተናል  5. ባለቤቴ በህይወት የለም |
| SD05 | የትምህርት ደረጃ | 1.ማንበብ እና መፃፍ የማይችሉ  2.ማንበብ እና መፃፍ የሚችሉ  3. አንደኛደረጃ  4.ሁለተኛ ደረጃእና የመሰናዶ ትምህርት የወሰዱ  5.ኮሌጅ / ዩኒቨርሲቲ / የሙያ ትምህርት የወሰዱ |
| SD06 | የስራ ሁኔታ | 1. ሥራአጥ  2. የመንግስትሰራተኛ  3. መንግስታዊያልሆነድርጅትሰራተኛ  4. የግልስራ |
| SD07 | አማካይወርሃዊገቢ | ______________ብር |
| SD08 | የቤተሰብ ብዛት | ______________ |
| SD09 | በቤትዎአቅራቢያ የፍራፍሬእናአትክልት ገበያአለ? | 1.አዎን 2.የለም |
|  |  |  |
| **ክፍል II- የጤንነት ሁኔታን የተመለከቱ ጥያቄዎች** | | |
|  |  |  |
| CC01 | የስኳር በሽታው አይነት(ከታካሚው) | 1. ዓይነት 1  2.ዓይነት 2  3. አላውቅም |
| CC02 | የስኳር በሽታው በምርመራከተረጋገጠ ስንት ግዜ ሆነው (ከአንድ አመት በታች ከሆነ በወር ተቀይሮ ይቀመጥ) | _______________ አመት/ወር |
| CC03 | እየወሰዱ ያሉት የመድሀኒትዓይነት | 1.መርፌ  2.በእንክብል መልክ(የሚዋጥ ክኒን)  3.ሁለቱንም ዓይነት  4.ምንም ዓይነት መድሃኒት አልወስድም |
| CC04 | ተያያዥ(ከስኳር ጋር ተጓዳኝ) በሽታዎች አሉቦት (የልብ የኩላሊት የነርቭ) (ከካርድ/መዝገብ) | 1. አዎን  2. አይ |
| CC05 | ስለ ስኳርህመምትምህርትይከታተላሉ | 1. ፈጽሞ  2. አዎንአንዳንድጊዜ  3. አዎንበመደበኛነት |
| CC06 | የስኳርህመምተኞችማህበርአባልነዎት | 1. አዎን  2.አይደለሁም |
| CC07 | በቤተሰብዎውስጥየስኳርህመምተኛየሆነሰውአለ? | 1. አዎን  2.የለም |
| CC08 | በቤትዎውስጥየራስዎግሉኮሜትር (የስኳር መጠኖን መለኪያ መሳሪያ)አለዎት | 1. አዎን  2. የለኝም |

**ክፍል III: ስኳርህመምየግልእንክብካቤተግባራትመጠይቅ**

ከዚህበታችየተዘረዘሩትጥያቄዎችባለፉትሰባትቀናትውስጥስለስኳርህመምዎየግልእንክብካቤተግባራትንበተመለከተምንእንደሚመስልየሚጠይቁናቸው፡፡ሆኖምግንባለፉት 7 ቀናትውስጥታመውከነበሩናእራስዎንበራስዎመንከባከብካልቻሉተጨማሪ 7 ቀናትወደኋላበመሄድጤነኛበነበሩበትጊዜያደረጉትእንክብካቤሁኔታመውሰድይችላሉ፡፡

|  | **ጥያቄዎች** | **የቀናትብዛትአማራጭመልሶች** | | | | | | | |
| --- | --- | --- | --- | --- | --- | --- | --- | --- | --- |
|  | **አመጋገብንበተመለከተ** | **0** | **1** | **2** | **3** | **4** | **5** | **6** | **7** |
| 301 | ባለፉት 7 ቀናትውስጥምንያህሌቀን/ናትነውጤነኛአመጋገብእቅድየነበርዎት? |  |  |  |  |  |  |  |  |
| 302 | ምንያህሌቀን/ናትበሳምንትውስጥይህንየአመጋገብእቅድዎንይከተሉ ነበር? |  |  |  |  |  |  |  |  |
| 303 | ባለፉት 7 ቀናትውስጥ በቀን 5 እናከዚያበላይጊዜአትክሌትናፍራፍሬምንያህሌቀን/ናት ተመግበዋል? |  |  |  |  |  |  |  |  |
| 304 | ባለፉት 7 ቀናተውስጥምንያህሌቀን/ናትከፍተኛየስብመጠንያለውምግብተመግበዋል/ለምሳሌ ቀይሥጋወይምበስብየተሞላየእንስሳትተዋጾኦ?(ፆምከመጀመሩበፊትያለውንጊዜይውሰዱ) |  |  |  |  |  |  |  |  |
| 305 | ባለፉት 7 ቀናትውስጥምንያህልቀን/ቀናትነውሀይልሰጪምግቦችንበቀንውስጥበእኩልበማመጣጠንየወሰዱት? |  |  |  |  |  |  |  |  |
|  | **የአካሌእንቅስቃሴማድረግበተመለከተ** | | | | | | | | |
| 306 | ባለፉት 7 ቀናትውስጥምንያህሌቀን/ናትለ30 ደቂቃያክልየአካልእንቅስቃሴኣድርገዋል (ሁለምእንቅስቃሴ፣ወክንጨምሮ፣ጠቅላላደቂቃ)? |  |  |  |  |  |  |  |  |
| 307 | ባለፉት 7 ቀናትውስጥምንያህሌቀን/ናትበተወሰኑየአካሌእንቅስቃሴተሳትፈዋል? (ቤትውስጥናስራቦታከሚያረጉትእንቅስቃሴውጭ) |  |  |  |  |  |  |  |  |
|  | **በደምየስኳርመጠንምርመራንማድረግበተመለከተ** | | | | | | | | |
| 308 | ባለፉት 7 ቀናትውስጥምንያህልቀን/ናትየስኳርመጠንምርመራአካሂደዋል? |  |  |  |  |  |  |  |  |
| 309 | ባለፉት 7 ቀናትውስጥምንያህልቀን/ናትየጤናባለሙያዎ/ሀኪሞበነገርዎትብዛትልክየስኳርመጠንምርመራኣድርገዋል? |  |  |  |  |  |  |  |  |
|  | **እግርናየእግርጣቶችእንክብካቤንበተመለከተ** | | | | | | | | |
| 310 | ባለፉት 7 ቀናትውስጥምንያህልቀን/ናትእግሮችዎንናየእግሮችዎንጣቶችመሀልፍተሻኣድርገዋል? |  |  |  |  |  |  |  |  |
| 311 | ባለፉት 7 ቀናትውስጥምንያህሌቀን/ናትየጫማዎን የውስጥክፍልምልከታኣድርገዋል? |  |  |  |  |  |  |  |  |
|  | **መድኃኒትን በተመለከተ** | | | | | | | | |
| 312 | ባለፉት 7 ቀናት ውስጥ ምን ያህሌ ቀን/ናት ነው የታዘዘሎትን መድኃኒት በትክክል የወሰዱት? |  |  |  |  |  |  |  |  |

**Part IV: diabetic health belief question**

ከዚህ በታች ያሉት አርፍተ ነገሮች ስለ ስኳር ህመምና የግል አንክብካቤ ያለዎትን ኣመለካከት መለኪያዎች ናቸው። በጥንቃቄ ካነበቡ በኋላ በአርፍተ ነገሩ ምን ያህል አንደሚስማሙ ወይም አንደማይስማሙ በመልስ መስጫ ሳጥኑ ውስጥ የ “√” ምልክት በማድረግ አመልክት/ቺ

1 = በደንብአልስማማም2 = አልስማማም3= ገለልተኛ 4= እስማማለሁ5= በደንብእስማማለሁ

| **ተ.ቁ.** | **የተጋላጭነት ግንዛቤ** | በደንብአልስማማም | አልስማማም | ገለልተኛ | እስማማለሁ | በደንብእስማማለሁ |
| --- | --- | --- | --- | --- | --- | --- |
| PT01 | የስኳርህመምተኛእንደመሆኔለተለያዩበሽታዎችተጋላጭ ነኝ (የኩላሊት፣ የልብ፣ የደም ግፊት እና ለመመሳሰሉት) |  |  |  |  |  |
| PT02 | የስኳርህመምተኛእንደመሆኔ በጊዜ ሂደት በተለያዩ በሽታወች ልጠቃ/ልያዝ እችላለሁ (የኩላሊት፣ የልብ፣ ለደም ግፊት እና ለመመሳሰሉት) |  |  |  |  |  |
| PT03 | የስኳርህመምተኛእንደመሆኔ በእግር ቁስለት / ጋንግሪን / የመጠቃትእድልአለኝ |  |  |  |  |  |
| PT04 | የስኳርህመምተኛእንደመሆኔ በደም ውሰጥ የግሉኮስ ማነስ (ሀይፖግላይሴሚያ)ሊያጋጥመኝይችላል |  |  |  |  |  |
| **የኣደገኝነት ግንዛቤ** | | | | | | |
| PT05 | እንደኩላሊት፣ልብእናየደምግፊትባሉበሽታዎችመጠቃትለስኳርህመምተኛከባድችግርነው፡፡ |  |  |  |  |  |
| PT06 | እንደኩላሊት፣ልብእናየደምግፊትባሉበሽታዎችመጠቃትለስኳርህመምተኛው ሕይወትአስጊነው |  |  |  |  |  |
| PT07 | በእግር ቁስለት (ጋንግሪን ) መጠቃት የስኳርህመምተኛን  የአካልክፍል ሊያሣጣይችላል |  |  |  |  |  |
| PT08 | የደም ውሰጥ ግሉኮስ ማነስ (ሃይፖግላይሴሚያ) የስኳርህመምተኛን ለድንገተኛሞትሊዳርግይችላል |  |  |  |  |  |
| **የጠቃሚነት ግንዛቤ** | | | | | | |
| PB01 | የስኳርበሽታአመጋገብጥሩስሜት/ጤንነትእንዲሰማኝያደርገኛል |  |  |  |  |  |
| PB02 | የአመጋገብልማዴንከቀየርኩምናልባትሊረዳኝይችላል |  |  |  |  |  |
| PB03 | የስኳርበሽታ አመጋገቤ በሽታዬን(የደም ውስጥ ሰኳሬን) እንደሚቆጣጠርአምናለሁ |  |  |  |  |  |
| **የትግበራ እንቅፋት ግንዛቤ** | | | | | | |
| PB04 | በስኳርበሽታአመጋገብላይያሉምግቦች የሚያስጠላ ጣዕምአላቸው |  |  |  |  |  |
| PB05 | ሐኪሙስለአመጋገብየነገረኝን (ያዘዘልኝን) ተግባራዊማድረግከባድነው |  |  |  |  |  |
| PB06 | ሐኪሜስለአመጋገቤየነገረኝሊገባኝአልቻለም፡፡ |  |  |  |  |  |
| PB07 | የሚመከረውንአመጋገብመከተልየተለመደውንየዕለትተዕለትእንቅስቃሴዬን የሚያስተጓጉልነው፡፡ |  |  |  |  |  |
| **የራስ ውጤታማነት** | | | | | | |
| SE01 | የስኳርህመምተኛ እንደመሆኔ ሊያጋጥሙኝ የሚችሉ፣እንደኩላሊት፣ልብእናየደምግፊትያሉበሽታዎችንለመከላከል የሚረዱ ምግቦችን (እንደአትክልቶች፣ፍራፍሬዎች፣ዝቅተኛጨውወዘተ)መመገብለእኔቀላልነው፡፡ |  |  |  |  |  |
| SE02 | የስኳርህመምተኛ እንደመሆኔ ሊያጋጥሙኝ የሚችሉ፣እንደኩላሊት፣ልብእናየደምግፊትያሉበሽታዎችንለመከላከል የሚረዱ ምግቦችን (እንደአትክልቶች፣ፍራፍሬዎች፣ዝቅተኛጨውወዘተ)መመገብ እንደምችል እርግጠኛ ነኝ |  |  |  |  |  |
| SE03 | በአካባቢዬያሉሰዎችየስኳርበሽታእንዳለብኝየማያውቁቢሆንም እንኳንበምግብዕቅዴላይመቆየትእንደምችልእርግጠኛነኝ፡፡ |  |  |  |  |  |
| SE04 | በየቀኑበተመሳሳይሰዓትምግብመብላትእንደምችልእርግጠኛነኝ |  |  |  |  |  |
| SE05 | ደስተኛነትወይምቁጡነትበሚሰማኝጊዜከመጠንበላይመብላትወይምአለመመገብመተውእንደምችልእርግጠኛነኝ፡፡ |  |  |  |  |  |

|  |  | 1. አዎ | 2. አይ |
| --- | --- | --- | --- |
| CA01 | የተወሳሰበ የስኳር ህመም ያለበት የቤተሰብ ኣባል አለህ/ሽ? |  |  |
| CA02 | ባለፈው 1 ወር ውስጥ የሚመከሩ የግል እንክብካቤ የሚተገብር ሰው ኣይተህ/ሽ ወይም ሰምተህ/ሽ ታውቃለህ/ቂያለሽ? |  |  |
| CA03 | ባለፈው 1 ወር ውስጥ የተወሳሰበ የስኳር ህመም ያለበት ሰው ኣይተህ/ሽ ወይም ሰምተህ/ሽ ታውቃለህ/ቂያለሽ? |  |  |
| CA04 | ባለፈው 1 ወር ውስጥ በመገናኛ ብዙሃን/ጋዜጣ የሚመከሩ የግል እንክብካቤ ስለመከተል ሰምተህ/ሽ ታውቃለህ/ቂያለሽ? |  |  |
| CA05 | ከሆስፒታሉ ስለ የስኳር ህመም የሚያስታውስ ፖስተር ወይም በራሪ ወረቀት ተሰቶህ ያውቃል |  |  |

**ክፍል V፡ ማህበረሰባዊ ድጋፍ**

አሁን ከቤተሰብ፣ ክጓደኛ አና ከለሎች ሰዎች ስለምታገኘው/ኚው ድጋፍ አጠይቆታለሁ። አርፍተ ነገሩን በደንብ በመከታተል ምን ያህል አንደሚስማሙ ወይም አንደማይስማሙ በመልስ መስጫ ሳጥኑ ውስጥ የ “√” ምልክት በማድረግ አመልክቱ

|  | መግለጫ | ምላሽ | | | | |
| --- | --- | --- | --- | --- | --- | --- |
| ተ.ቁ |  | በደንብ አልስማማም | አልስማማም | ገለልተኛ | እስማማለሁ | በደንብ እስማማለሁ |
|  | **ቤተሰብ** |  |  |  |  |  |
| 1 | ከቤተሰቦቼ የሞራል ድጋፍና አገዛ አገኛለሁ |  |  |  |  |  |
| 2 | ቤተሰቦቼ የእውነትም አኔን ለመርዳት ይጥራሉ |  |  |  |  |  |
| 3 | ቤተሰቦቼ ውሳኔ አንድወስን ለመርዳት ፍላጎት ኣላቸው |  |  |  |  |  |
| 4 | ስለ ችግሮቼ ከቤተሰቦቼ ጋር ማውራት አችላለሁ |  |  |  |  |  |
|  | **ጓደኞች** |  |  |  |  |  |
| 5 | ጓደኞቼ የእውነትም አኔን ለመርዳት ይጥራሉ |  |  |  |  |  |
| 6 | ነገሮች ጥሩ ሳይሆኑ ሲቀር ሊረዱኝ የሚችሉ ጓደኞቼን መቁጠር አችላለሁ |  |  |  |  |  |
| 7 | ስለ ችግሮቼ ከጓደኞቼ ጋር ማውራት አችላለሁ |  |  |  |  |  |
| 8 | ደስታና ሃዘኔን ሊጋሩኝ የሚችሉ ጓደኞች ኣሉኝ |  |  |  |  |  |
|  | **ሌሎች** |  |  |  |  |  |
| 9 | በ ህይወቴ ውስጥ ስለ ስሜቴ የሚጨነቅ የተለየ ሰው ኣለ |  |  |  |  |  |
| 10 | በ ህይወቴ ውስጥ የአውነተኛ ምቾት ምንጭ የሆነ የተለየ ሰው ኣለ |  |  |  |  |  |
| 11 | በ ህይወቴ ውስጥ በሚያስፈልገኝ ሰአት ከአጠገቤ ማገኘው የተለየ ሰው ኣለ |  |  |  |  |  |
| 12 | በ ህይወቴ ውስጥ ደስታና ሃዘኔን ሊጋራኝ የሚችል የተለየ ሰው ኣለ |  |  |  |  |  |

**ክፍል VI፡ የስኳርህመምእናራስንመንከባከብየእውቀትጥያቄ**

ይሄ ስለ ስኳር በሽታና የግል አንክብካቤ መጠየቂያ ነው። ጥያቄውንና ምርጫውን በትኩረት ከተከታተሉ በኋላ ትክክል ነው በሚሉት ምርጫ ላይ ያክብቡ “0” ምልክት ያድርጉ።

| K01 | ለስኳርህመምተኛየሚመከሩምግቦችየትኞቹናቸው | |
| --- | --- | --- |
|  | ሀ. አብዛኛውኢትዮጲያዊየሚመገበውምግብ | ለ. የተመጣጠነምግብ |
|  | ሐ. ለ አብዛኛው ሰው ብዙየስኳርመጠንያለውምግብ | መ. ለ አብዛኛው ሰው ብዙየፕሮቲንመጠንያለውምግብ |
| K02 | ከሚከተሉትውስጥብዙየስኳርመጠንያለውምግብየቱነው | |
|  | ሀ. የበሰለ የዶሮ ስጋ | ለ. እርጎ |
|  | ሐ. የበሰለድንች | መ. የለውዝቂቤ |
| K03 | ከሚከተሉትውስጥከፍተኛየቅባትመጠንያለውምግብየቱነው | |
|  | ሀ. ወተት | ለ. የብርቱካንጭማቂ |
|  | ሐ. በቆሎ | መ. ማር |
| K04 | ከሚከተሉት ውስጥ ከስኳር ነፃ የሆነው ምግብ የቱ ነው | |
|  | ሀ. ማንኛውም የማይጣፍጥ ምግብ | ለ. ማንኛውም የስኳር ህመምተኛ ምግብ |
|  | ሐ. ስኳር የለውም ተብሎ የተፃፈበት ምግብ | መ. ማንኛውም ትንሽ የስኳር መጠን በውስጡ የያዘ ምግብ |
| K05 | በዛሬው እለት የተለኩት የስኳር መጠን ውጠት የሚያሳየው የየትኛውን ግዜ ነው | |
|  | ሀ. የ1 ቀን ነው | ለ. የ 1 ሳምንት ነው |
|  | ሐ. የ6 ሳምንት ነው | መ. የ 6 ወር ነው |
| K06 | ከሚከተሉት ስኳርን መመርመሪያ ዘዴዎች ውስጥ የተሻለ የስኳር መጠንን የሚያሳውቀው የትኛው የምርመራ አይነት ነው | |
|  | ሀ. የሽንት ምርመራ | ለ. የደም ምርመራ |
|  | ሐ. ሁለቱም ጥሩ ናቸው |  |
| K07 | የማይጣፍጥ የአትክልት ጭማቂ በደም ውስጥ ያለውን የስኳር መጠን | |
|  | ሀ. ይቀንሳል | ለ. ይጨምራል |
|  | ሐ. ምንም ለውጥ አያመጣም |  |
| K08 | ከሚከተሉት ምርጫ ውስጥ የደም ስኳር መጠን ሲያንስብን የማንጠቀመው የትኛውን ነው? | |
|  | ሀ. 3 ደረቅ ከረሜላ | ለ. ግማሽ ኩባያ ብርቱካን ጭማቂ |
|  | ሐ. 1 ኩባያ ለስላሳ መጠጥ | መ. አንድ ኩባያ ወተት |
| K09 | የስኳር መጠኑ የተስተካከለለት የስኳር ህመምተኛ የአካል እንቅስቃሴ ቢያደርግ በደሙ ውስጥ ያለውን የስኳር መጠን ምን ይሆናል፡ | |
|  | ሀ. ይቀንሳል | ለ. ይጨምራል |
|  | ሐ. ምንም ለውጥ አያመጣም |  |
| K10 | የኢንፌክሽን ህመም በደም ስኳር መጠን ላይ ሊያስከትል የሚችለው ችግር ምድነው | |
|  | ሀ. የደም ስኳር መጠንን ይጨምራል | ለ. የደም ስኳር መጠንን ይቀንሳል |
|  | ሐ. የደም ስኳር መጠን ላይ ምንም ለውጥ አያመጣም |  |
| K11 | ከሚከተሉት ውስጥ የእግሮውን ጤና ለመጠበቅ የተሻለ ዘዴ የትኛው ነው | |
|  | ሀ. በየቀኑ እግርን ማየትና መታጠብ | ለ. በየቀኑ እግርን በአልኮል ማሸት |
|  | ሐ. በየቀኑ ለ1 ሰዓት እግርን በውሃ ውስጥ መዘፍዘፍ | መ. ከወትሮው የሚተልቅ ቁጥር ያለውን ጫማ ማድረግ |
| K12 | በውስጡ አነስተኛ የቅባት መጠን ያለውን ምግብ መመገብ ከምን ይከላከላል | |
|  | ሀ. ከነርቭ በሽታ | ለ. ከኩላሊት በሽታ |
|  | ሐ. ከልብ በሽታ | መ. ከአይን በሽታ |
| K13 | የእጅ /የእግር መደንዘዝ/ ማቃጠል ስሜት የምን ምልክት ነው | |
|  | ሀ. የኩላሊት በሽታ | ለ. የነርቭ በሽታ |
|  | ሐ. የአይን በሽታ | መ. የጉበት በሽታ |
| K14 | ከሚከተሉት ውስጥ አብዛኛውን ጊዜ ከስኳር ህመም ጋር የማይያያዘው ቸግር የትኛው ነው) | |
|  | ሀ. የአይን ችግር | ለ. የኩላሊት ችግር |
|  | ሐ. የነርቭ ችግር | መ. የሳምባ ችግር |

**ክፍል VII፡ ከስኳር ጋር የተያያዘ ጭንቀትን በተመለከተ**

ከታች ከስኳር ጋር የተያያዘ ጭንቀትን በተመለከተ 17 ጥያቄዎች አሉ።በደንብ በማድመጥ አስተያየቶቹ (አ/ነገሩ) እርሶ ዘንድ በምን ያህል መጠን ችግር እንደሆነ በ“√” ያመልክቱ።

1= ችግር ኣይደለም 2= በጥቂቱ ችግር ነው 3= መካከለኛ ችግር ነው 4= በመጠኑ ኣሳሳቢ ችግር ነው 5= ኣሳሳቢ ችግር ነው 6= በጣም ኣሳሳቢ ችግር ነው

| ተቁ | መግለጫ | ምላሽ | | | | | |
| --- | --- | --- | --- | --- | --- | --- | --- |
|  |  | 1 | 2 | 3 | 4 | 5 | 6 |
| 701 | የስኳር በሽታ ኣብዛኛውን ጊዜየን የተሻማኝ ኣንደሆነ ይሰማኛል |  |  |  |  |  |  |
| 702 | ዶክተሬ ስለ ስኳር በሽታ ኣና ስለ ግል አንከበካቤ በደንብ ኣያውቅም ብየ ይሰማኛል |  |  |  |  |  |  |
| 703 | ከስኳር በሸታ ጋር ስለመኖር ሳስብ ንዴት፣ ጭንቀትና ድብርት ይሰማኛል |  |  |  |  |  |  |
| 704 | ዶክተሬ የስኳር በሸታን አንዴት መቆጣጠር አንዳለብኝ ግል መንገድ አንዳልነገረኝ ይሰማኛል |  |  |  |  |  |  |
| 705 | የደም ስኳር መጠኔን በበቂ ሁኔታ በተደጋጋሚ አየለካሁ አንዳልሆነ ይሰማኛል |  |  |  |  |  |  |
| 706 | የተለመደውን የስኳር በሽታ እንክብካቤ መተግበር ላይ አንዳልተሳካልኝ ይሰማኛል |  |  |  |  |  |  |
| 707 | ቤተሰቤ ወይም ጓደኞቼ የግል ክብካቤ ጥረቴን በበቂ መጠን እንዳልደገፉኝ ይሰማኛል( ለምሳሌ ከኔ እቅድ ጋር የሚጋጭ እቅድ ማውጣት፣ትክክል ያልሆነ ምግብ እንድመገብ ማበረታታት) |  |  |  |  |  |  |
| 708 | የስኳር በሽታ ህይወቴን አንደሚቆጣጠረው ይሰማኛል |  |  |  |  |  |  |
| 709 | ዶክተሬ ጭንቀቴ አንዳላሳሰበው ይሰማኛል |  |  |  |  |  |  |
| 710 | ቀን በቀን የስኳር በሸታን ለመቆጣጠር አችላለሁ ብዬ ኣይሰማኝም |  |  |  |  |  |  |
| 711 | ምንም ነገር ባደርግ የረዥም ጊዜ የተወሳሰበ የሰኳር በሽታ አንደሚይዘኝ ይሰማኛል |  |  |  |  |  |  |
| 712 | ጥሩ የሆነ የኣመጋገብ አቅድ በደንብ አየተገበርኩ አንዳልሆነ ይሰማኛል |  |  |  |  |  |  |
| 713 | ቤተሰብና ጓደኞቼ ከሰኳር በሽታ ጋር መኖር ምን ያህል ከባድ አንደሆነ ኣይረዱም ብዬ ይሰማኛል |  |  |  |  |  |  |
| 714 | ከስኳር በሽታ ጋር ለመኖር በሚያስፈልጉ ነገሮች የመዳከም ስሜት ይሰማኛል |  |  |  |  |  |  |
| 715 | ከስኳር በሽታዬ ጋር በተያያዘ በቋሚነት ላገኘው የምችለው ዶክተር አንደሌለኝ ይሰማኛል |  |  |  |  |  |  |
| 716 | የስኳር በሽታን የግል አንክብካቤ ለመከታተል ተነሳሽነት ኣይሰማኝም |  |  |  |  |  |  |
| 717 | ቤተሰቤና ጓደኞቼ የምወደውን የ,,,,,,,,,,,,,,,,,,ድጋፍ ኣይሰጡኝም ብዬ ይሰማኛል |  |  |  |  |  |  |

**ክፍል VIII :- ባህሪን የተመለከቱ ጥያቄዎች**

| ተ.ቁ | ጥያቄ | ምላሽ | አስተያየት |
| --- | --- | --- | --- |
| D01 | አጭሰው ያውቃሉ | 1. አዎ 2. አይ | ኣይ ከሆነ ወደ D05 |
| D02 | በአሁን ጊዜ ያጨሳሉ (ሲጋራ፣ ሺሻ) | 1. አዎ 2. አይ |  |
| D03 | ለጥያቄ D02 መልሶ አዎ ከሆነ, በአሁን ጊዜ በየቀኑ ያጨሳሉ? | 1. አዎ 2. አይ |  |
| D04 | በሳምንት ውስጥ ምን ያህል ቀን ያጨሳሉ? | _____ቀን |  |
| D05 | ባለፉት 12 ወራት ጫት ቅመው ያውቃሉ? | 1. አዎ 2. አይ | ኣይ ከሆነ ወደ D07 |
| D06 | አዎ ከሆነ, በሳምንት ውስጥ ምን ያህል ቀን ይቅማሉ? | _____ቀን |  |
| D07 | የአልኮል መጠጥ ጠጥተው ያውቃሉ/ በፊት ወይም አሁን/? (ቢራ፣ጠላ፣ኣረቄ፣ወይን ሌላም) | 1. አዎ 2. አይ | አይ ከሆነ ወደ ቀጣዮቹን ጥያቄዎች ይለፏቸው |
| D08 | ባለፉት 12 ወራት የአልኮል መጠጥ ጠጥተው ያውቃሉ? | 1. አዎ 2. አይ |  |
| D09 | ለጥያቄ አዎ ከሆነ, ቢያንስ አንድ የአልኮል መጠጥ ምን ያህል ጊዜ ይጠጣሉ? | 1. በየቀኑ 2. በሳምንት ከ5-6 ቀን 3. በሳምንት ከ1-4 ቀን 4. በወር ከ1-3 ቀን 5. በወር ከ አንድ ቀን ያነሰ |  |
